# Genome-Wide Transcriptional Changes of *Rhodosporidium kratochvilovae* at Low Temperature

**DOI:** 10.3389/fmicb.2021.727105

**Published:** 2021-09-16

**Authors:** Rui Guo, Meixia He, Xiaoqing Zhang, Xiuling Ji, Yunlin Wei, Qi-Lin Zhang, Qi Zhang

**Affiliations:** Faculty of Life Science and Technology, Kunming University of Science and Technology, Kunming, China

**Keywords:** *Rhodosporidium kratochvilovae*, low temperature, genome sequencing, differential expressed genes, RNA-Seq, qPCR

## Abstract

*Rhodosporidium kratochvilovae* strain YM25235 is a cold-adapted oleaginous yeast strain that can grow at 15°C. It is capable of producing polyunsaturated fatty acids. Here, we used the Nanopore Platform to first assemble the *R. kratochvilovae* strain YM25235 genome into a 23.71 Mb size containing 46 scaffolds and 8,472 predicted genes. To explore the molecular mechanism behind the low temperature response of *R. kratochvilovae* strain YM25235, we analyzed the RNA transcriptomic data from low temperature (15°C) and normal temperature (30°C) groups using the next-generation deep sequencing technology (RNA-seq). We identified 1,300 differentially expressed genes (DEGs) by comparing the cultures grown at low temperature (15°C) and normal temperature (30°C) transcriptome libraries, including 553 significantly upregulated and 747 significantly downregulated DEGs. Gene ontology and pathway enrichment analysis revealed that DEGs were primarily related to metabolic processes, cellular processes, cellular organelles, and catalytic activity, whereas the overrepresented pathways included the MAPK signaling pathway, metabolic pathways, and amino sugar and nucleotide sugar metabolism. We validated the RNA-seq results by detecting the expression of 15 DEGs using qPCR. This study provides valuable information on the low temperature response of *R. kratochvilovae* strain YM25235 for further research and broadens our understanding for the response of *R. kratochvilovae* strain YM25235 to low temperature.

## Introduction

One of the crucial ecological factors associated with abiotic stress is low temperatures, which can disrupt microbial homeostasis and affect the biological functions of cells (García-Ríos et al., 2017; Wang et al., 2020). In addition, low temperatures can severely inhibit fungal growth and may also kill it (Wang et al., 2014; Long et al., 2015). Although 10–18°C is considered as a low temperature range for fungus, these are permissive temperature (García-Ríos et al., 2017). Currently, the understanding of the molecular mechanisms of response in fungus to low temperature stress has been explored. For example, several fungal genera adapt to low temperature by changing the expression of different genes as part of the process known as the cold-shock response that has been investigated in *Saccharomyces cerevisiae* (Aguilera et al., 2007). During adaptation, several cold response-related genes, including those involved in energy preservation, detoxification, osmolyte production, protein folding support, and maintenance of membrane fluidity, are activated (Murata et al., 2006). Growth capacity diminishes as the temperature decreases below the optimum temperatures. Studies in *Neurospora crassa* have demonstrated a high accumulation of mRNAs of carotenoid genes to reduce oxidative stress under low temperature (Castrillo et al., 2018). These results suggest that differentially expressed genes (DEGs) play a key role under low temperature stress in fungi. Therefore, the identification of genes associated with low temperature response and intensive exploration for molecular mechanisms in response to low temperature at the level of gene expression in fungi are necessary.

*Rhodosporidium* species have been reported to be capable of synthesizing some value-added compounds with a wide industrial usage, such as lipid (Uprety et al., 2017), carotenoids (Buzzini et al., 2007), enzymes (MacDonald et al., 2016), polyunsaturated fatty acids (PUFAs) (Ageitos et al., 2011; Cui et al., 2016; Wang et al., 2017; He et al., 2019) and sugar alcohols including D-arabitol and galactitol (Jagtap and Rao, 2018; Jagtap et al., 2019). As a species of the genus *Rhodosporidium*, *Rhodosporidium kratochvilovae* strain YM25235 (isolated from Chenghai Lake, Yunnan, China) is a cold-adapted oleaginous fungal yeast strain that can grow at 15°C (He et al., 2019), and it is capable of producing PUFAs (Cui et al., 2016; Wang et al., 2017; He et al., 2019). PUFAs are involved in the maintenance of optimal physical and biological properties of cell membranes, which is an essential adaptation strategy in microorganisms against cold stress (Clarke, 2001; Sampath and Ntambi, 2004). Certain fungi produce high levels of PUFAs in response to low temperature (Calvo et al., 2001; Wang et al., 2017). We had previously reported that the strain YM25235 at 15°C produced high amounts of linoleic acid (LA, C18:2Δ9,12) and α-linolenic acid (ALA, C18:3Δ9,12,15), and the inhibition of PUFA biosynthesis negatively influenced the cold adaptation of YM25235 (Cui et al., 2016; Wang et al., 2017). In addition, cold-adapted fungal yeasts have attracted the wide attention of several scientists worldwide because of their significant potential for application in diverse industries (Margesin and Miteva, 2011; Carrasco et al., 2017). These fungal species have evolved physiological strategies to survive in cold climates, including modulation of enzyme kinetics and membrane fluidity (Firdaus-Raih et al., 2018).

For *R. kratochvilovae*, Miccoli et al. (2018) have completed whole genome sequencing of the strain LS11, and several other *Rhodosporidium* strains have also been sequenced (Hu and Ji, 2016; Zhang et al., 2016; Tran et al., 2019). In addition, multi-omics analyses of the oleaginous fungal yeast *Rhodosporidium toruloides* were conducted to shield lights on Pi-limitation-induced lipid accumulation (Wang et al., 2018). By comparing microbial growth and gene expression patterns, several studies demonstrated that expression levels of genes that encode carotenoid biosynthesis were altered in response to light change (Pham et al., 2020). Dinh et al. (2019) reconstructed a genome-scale model of *R. toruloides* IFO0880’s metabolic network. However, changes in the gene expression profile of these fungal yeasts, especially *R. kratochvilovae*, under temperature stress have not been investigated.

We performed this study to gain a comprehensive understanding of the molecular adaptation mechanisms to low temperatures in *R. kratochvilovae* strain YM25235 from the perspective of gene expression. The whole genome of *R. kratochvilovae* strain YM25235 was sequenced. Subsequently, based on RNA-seq data and the above-sequenced genome of strain YM25235, DEGs were identified between *R. kratochvilovae* at low temperature (15°C) and normal temperature (30°C) groups. Furthermore, we performed annotation of *R. kratochvilovae* strain YM25235 genes and functional enrichment analysis [Gene Ontology (GO) and Kyoto Encyclopedia of Genes and Genomes (KEGG) pathway] of DEGs. The DEGs were summarized, and several key pathways involved in low temperature stress tolerance were obtained from the list of KEGG terms enriched by the DEGs. This study will provide insights into the molecular adaptation mechanisms of *R. kratochvilovae* strain YM25235 under low temperature stress.

## Materials and Methods

### Culture Conditions and Cell Sample Preparation

For whole genome sequencing, *R. kratochvilovae* strain YM25235 was grown in yeast extract peptone dextrose (YPD: 1% yeast extract, 2% peptone, and 2% glucose) broth at 30°C to logarithmic growth phase (OD_600_ = 1.60). For RNA-seq, *R. kratochvilovae* strain YM25235 was pre-incubated in YPD broth at 30°C for 24 h and then was exposed to 15 and 30°C for another 8 h, with the final OD_600_ values of ∼2.1 and ∼2.3, respectively, with three technical replicates collected for each sample. The culture broth was centrifuged at 5,000 × *g* for 10 min at 4°C. For cell disruption, the culture pellets were ground in liquid nitrogen.

### DNA Extraction, Genome Sequencing, and Assembly

Total DNA of *R. kratochvilovae* strain YM25235 was extracted using Wizard Genomic DNA Purification Kit (Promega, Madison, WI, United States). The next experimental procedures were performed according to the standard protocols provided by Oxford Nanopore Technologies (ONT). To optimize the sequencing experiments and improve the throughput, a library was constructed using the SQK-LSK109 Ligation Sequencing Kit. Then, the library was sequenced on the Nanopore PromethION platform. After sequencing, the downstream sequencing data were analyzed by basecalling programs using the Albacore software from the MinKNOW package to convert the raw sequencing data from FAST5 format to FASTQ format (Wick et al., 2019). Further filtering for the adaptor, low quality, and short reads (<2,000 bp in length) resulted in total dataset clean reads. Canu v1.5 software was used to correct the filtered subreads (Koren et al., 2017). Next, we assembled the subreads after error correction using the wtdbg software to obtain the final genome with high accuracy (Ruan and Li, 2019). The BUSCO v2.0 software was used to assess the completeness of the *R. kratochvilovae* strain YM25235 genome assembly (Simão et al., 2015).

### Functional Annotation of the Genome

Because of the relatively low conservation of repeat sequences among different species, predictions of repeat sequences for a particular species require the construction of specific repeat sequence databases. Therefore, we used four software, including LTR_FINDER v1.05, MITE-Hunter, RepeatScout v1.05, and PILER-DF v2.4, to construct a repeat sequence database for the genome of *R. kratochvilovae* strain YM25235 based on the methods of structural and *de novo* predictions (Edgar and Myers, 2005; Price et al., 2005; Xu and Wang, 2007; Han and Wessler, 2010). Classification of the repeat sequences was performed using the PASTE Classifier software (Wicker et al., 2007). The type of the classified repeat sequences was subsequently annotated using the Repbase database (Jurka et al., 2005). Repeat sequences in the genome of *R. kratochvilovae* strain YM25235 were searched in the RepeatMasker v4.0.6 database (Chen, 2004).

The gene structure was predicted using *de novo* predictions and homologous protein search. Transcript evidence from sequencing transcriptome was used to validate the coding regions of genes. Finally, the prediction results from the three methods were intersected as the final sets of protein-coding genes. In particular, *de novo* prediction was performed using GenScan, Augustus v2.4, GlimmerHMM v3.0.4, GeneID v1.4, SNAP (version 2006–07–28) (Burge and Karlin, 1997; Stanke and Waack, 2003; Korf, 2004; Majoros et al., 2004; Alioto et al., 2018). Homologous protein-based predictions were implemented using GeMoMa v1.3.1 (Keilwagen et al., 2016); and transcript-based predictions were conducted using the PASA v2.0.2 software (Campbell et al., 2006). The intersection of the results from three methods was obtained by using EVM v1.1.1 (Haas et al., 2008).

To obtain functional annotation information of *R. kratochvilovae* strain YM25235 genes, the predicted gene sequences were annotated by searching them in the following gene collections using the BLAST tool: Cluster of orthologous groups for eukaryotic complete genomes (KOG) (Tatusov et al., 2000), KEGG (Kanehisa et al., 2004), Swiss-Prot (Boeckmann et al., 2003), and non-redundant protein sequence database (Nr) (Deng et al., 2006). Based on the searched results from the Nr database, the GO annotation information of genes was extracted using the in-house Perl script (Ashburner et al., 2000; Conesa et al., 2005). In addition, domains of the protein sequence of each gene were searched in the Pfam database using the Hmmer software (Eddy, 1998; Finn et al., 2016).

### Extraction of RNA, Library Construction, and Sequencing

Total RNA of *R. kratochvilovae* strain YM25235 was purified using RNeasy Mini Kit (Qiagen, Valencia, CA, United States). After extraction of total RNA from the samples, the quality of total RNA [i.e., RNA integrity number (RIN) values, 28S/18S and concentration] was determined using the Agilent 2100 Bioanalyzer (Agilent RNA 6000 Nano Kit). The purity of the samples was assessed using a UV spectrophotometer NanoDrop^TM^, ND-1000 (Thermo Scientific, United States). Next, the cDNA library was constructed. In brief, *R. kratochvilovae* strain YM25235 mRNA was enriched using magnetic beads with oligo (dT). The resulting mRNA was fragmented by adding an appropriate amount of the shearing reagent under high temperature conditions so that the interrupted mRNA was used as a template for the first-strand cDNA synthesis. Subsequently, the second strand cDNA was synthesized, purified using commercial kits, and sticky ends were repaired. The base “a” was added to the 3′ end of the cDNA and adaptors were ligated, followed by fragment size selection and PCR amplification. The quality of the constructed libraries was checked using the Agilent 2100 Bioanalyzer (Agilent Technologies, United States) and ABI StepOnePlus Real-Time PCR System.

Raw reads generated by RNA sequencing (RNA-seq) were filtered to obtain clean data by removing low-quality reads, adapter contamination, and reads with excessive unknown bases (Cock et al., 2010). Clean reads were mapped to the reference genome of *R. kratochvilovae* strain YM25235 to avoid contaminants caused by non-*R. kratochvilovae* strain YM25235 reads using Hierarchical Indexing for Spliced Alignment of Transcripts (HISAT) (Kim et al., 2015).

### Quantification of Gene Expression and Identification of Differentially Expressed Genes

Clean reads were mapped to the reference gene sequences using Bowtie 2 (Langmead and Salzberg, 2012). Next, the expression of genes was assessed based on Fragments Per Kilobase per Million (FPKM) values calculated by RSEM (RNA-Seq by Expectation Maximization) methods (Li and Dewey, 2011). DEGs were identified using the DESeq2 software, an R program suitable for the identification of DEGs from high-throughput sequencing data between the control and treatment groups (Love et al., 2014). The DEGs with fold change (FC) ≥ 2 (| log_2_ ratio| ≥ 1) and *p*-values (Wald test in Deseq2) corrected by a false discovery rate (corrected *p*-values) < 0.05 were identified as DEGs.

### Functional Enrichment Analysis of Differentially Expressed Genes

To further elucidate the biased biological function of DEGs and signaling pathways, the functional analyses of DEGs were performed using the Blast2GO pipeline (Conesa et al., 2005). The annotated functional terms of DEGs were clustered through the Functional Annotation Clustering tool in the KOBAS software (Xie et al., 2011). The KEGG pathways from each clustered set with an FDR-value < 0.01 were considered to be significantly enriched. Furthermore, the redundant GO terms were systematically discarded using GO trimming (Jantzen et al., 2011).

### Validation of RNA-Seq Data and Gene Expression Analysis by qPCR

To validate the results of RNA-seq, 15 DEGs were randomly selected for downstream quantitative real-time PCR (qPCR) analysis, with three biological replicates (Table 1). Parallelly, we obtained the corresponding control sample in the same manner. Purification and evaluation of total RNA were performed using the above-mentioned methods (“Extraction of RNA, Library Construction, and Sequencing”). This was performed thrice independently to generate three biological replicates.

**TABLE 1:** List of randomly selected 15 differentially expressed genes (DEGs).

Gene name	Log_2_FoldChange (Treatment/Control)	Gene symbol	Pathway	*P*_adj_
Aryl-alcohol dehydrogenase	–2.9209	AAD	MAPK signaling pathway-yeast	2.61E-53
LigA	2.5233	LigA	Amino sugar and nucleotide sugar metabolism	1.72E-10
Alcohol dehydrogenase	–3.6458	Adh	Amino sugar and nucleotide sugar metabolism	1.83E-117
Acetolactate synthase	1.6086	ALS	Butanoate metabolism	1.31E-22
GTP cyclohydrolase II	–5.0704	GCH2	NA	3.12E-115
4-Coumarate-CoA ligase	–2.0127	4CCL	NA	1.05E-07
Cell cycle checkpoint protein rad17	–1.4118	rad17	Cell cycle-yeast	4.50E-16
Sulfate permease	1.8478	SLP	NA	0.00126
Isocitrate lyase	–2.8528	ICL	Metabolic pathways	3.76E-28
Proteophosphoglycan ppg4	1.9823	ppg4	NA	1.25E-40
ATP phosphoribosyltransferase	2.0123	hisG	Metabolic pathways	2.62E-52
GCN5-related *N*-acetyltransferase	1.3899	GNAT	NA	9.88E-13
MFS multidrug transporter	–1.9306	MFSmdf	Amino sugar and nucleotide sugar metabolism	8.21E-33
Cytosine-purine permease	–2.4662	FCY	MAPK signaling pathway-yeast	6.64E-27
RTA-like protein	–1.9852	RTK	Amino sugar and nucleotide sugar metabolism	2.59E-44

Specific primers used in the qPCR analysis were designed automatically using the Beacon Designer 7 (Supplementary Table 1). For the preparation of cDNA libraries, the first-strand cDNA was synthesized from total RNA using the PrimeScript RT reagent kit with gDNA eraser (TaKaRa, Tokyo, Japan). Next, we used RNase-free water to dilute the preliminary cDNA solution (final concentration, 100 ng/μL). Next, qPCR was performed on the Light Cycler 480 Real-Time PCR System (Roche, Basel, Switzerland) using the Bestar SYBR Green qPCR Master mix (DBI Bioscience, Shanghai, China). The qPCR reaction of each gene in each sample was independently repeated thrice, and experiments were performed in three biological replicates. The qPCR amplification steps were a holding stage at 95°C for 5 min, followed by the cycling stage with 40 cycles of 94°C for 10 s and 60°C for 40 s. The subsequent melt curve stage consisted of 95°C for 15 s, 60°C for 1 min, and 95°C for 15 s. Small subunit rRNA (SSU rRNA) was used as an internal control. The relative expression of target genes was calculated by comparison with SSU rRNA using the 2^–ΔΔCt^ method. The final results are shown as means ± standard deviations (SD) (Livak and Schmittgen, 2001).

## Results

### Summary and Assembly Information of Genome Sequencing Data

A total of 4,795,537,118 bp raw data of *R. kratochvilovae* strain YM25235 were obtained from the Nanopore sequencer. Furthermore, 4,578,684,148 bp clean data were obtained. Sequencing depth was 193.04× and the total length of the assembled complete genome was 23.71 Mb, which is higher than that of *R. kratochvilovae* strain LS11 (22.11 Mb), *R. toruloides* strain NP11 (20.22 Mb), and *R. paludigena* (20.66 Mb). The N50 length of the assembly was 1,067,950 bp, which is higher than that of *R. kratochvilovae* strain LS11 (704,466 bp), *R. toruloides* strain NP11 (163,970 bp), and *R. paludigena* (371,695 bp). The average GC content of *R. kratochvilovae* strain YM25235 genome (67.30%) is higher than that of *R. kratochvilovae* strain LS11 (66.60%), *R. toruloides* strain NP11 (62.00%), and *R. paludigena* (64.3%). A total of 8,472 genes were identified in the genome of *R. kratochvilovae* strain YM25235, with an average length of 2,151 bp. The number of coding sequence (CDS) was 57,762, and the average length of CDS was 229.57 bp; the average number of CDS per gene was 6.82 bp. The number of introns was 50,188 bp and the average length of the intron was 82.44 bp. Each gene average contained 5.92 introns (Table 2). The results of BUSCO showed that 84.83% of the genes were completed (Table 3), indicating the high quality and completeness of the current genome (Jiang et al., 2019).

**TABLE 2:** Genome assembly results statistics.

**Item**	

**Scaffold features**	
Total number	46
Total length (bp)	23,718,404
N50 (bp)	1,067,950
N90 (bp)	255,572
Max length (bp)	2,421,707
Mean Qual	8.92
Sequence GC content (%)	67.30

**Genome features**	

Genome assembly (Mb)	18,229,638
Number of protein-coding genes	8,472
Average gene length (bp)	2,151.75
Average coding sequence length (bp)	229.57
Exon length (nt)	14,092,373
Average exon length (nt)	240.24
Exon number	58,660
Average exon number	6.92
intron length (nt)	4,137,265
Average intron length (nt)	82.44
intron number	50,188
Average intron number	5.92

**TABLE 3:** BUSCO evaluation statistics.

**Item**	
Complete BUSCOs(C)	246 (84.83%)
Complete and single-copy BUSCOs(S)	226 (77.93%)
Complete and duplicated BUSCOs(D)	20 (6.90%)
Fragmented BUSCOs(F)	21 (7.24%)
Missing BUSCOs(M)	23 (7.93%)
Total Lineage BUSCOs	290

### Gene Prediction and Annotation

**FIGURE 1:**
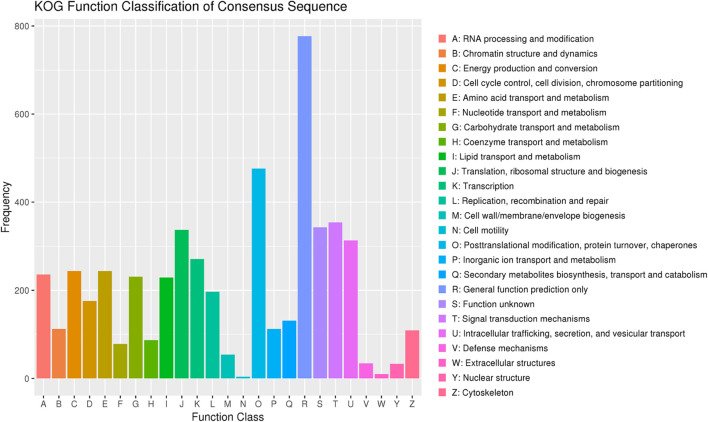
The Eukaryotic Orthologous Groups (KOG) classification of proteins in *Rhodosporidium kratochvilovae* strain YM25235. The distribution of predicted proteins from *R. kratochvilovae* strain YM25235 genome according to the functional class by KOG database.

**FIGURE 2:**
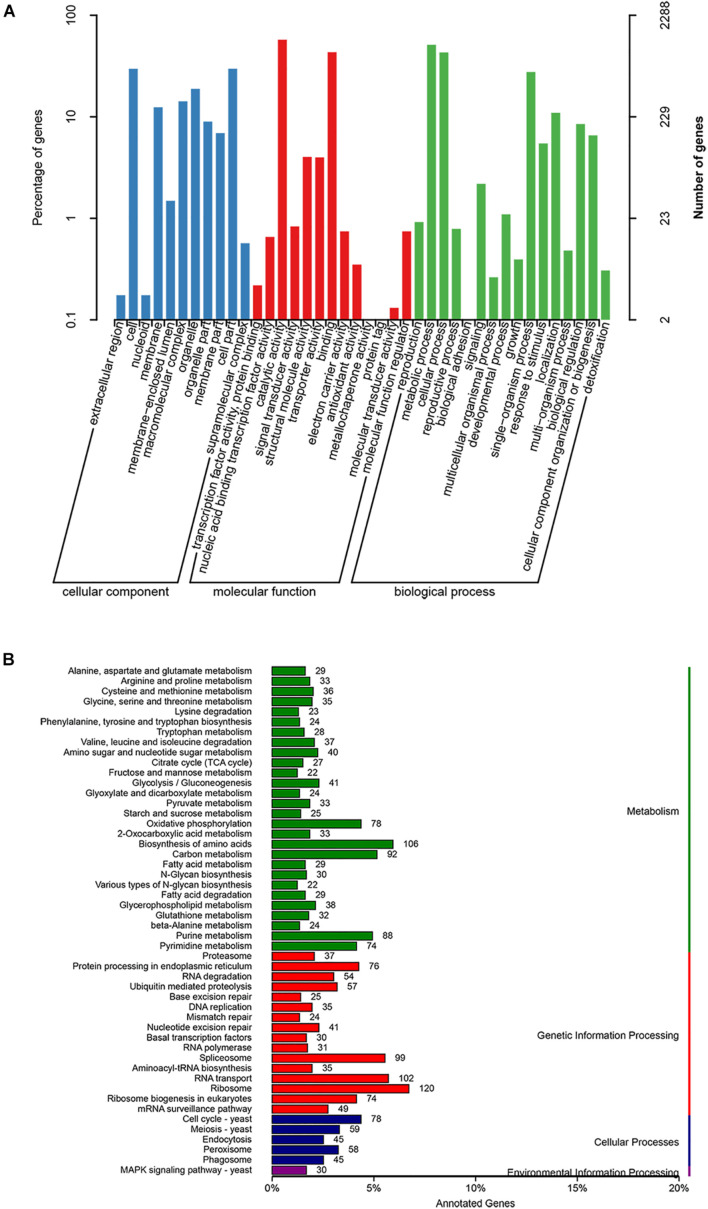
Gene ontology (GO) and Kyoto Encyclopedia of Genes and Genomes (KEGG) classifications of proteins in *R. kratochvilovae* strain YM25235. Distribution of predicted proteins from *R. kratochvilovae* strain YM25235 genome by **(A)** GO and **(B)** KEGG databases.

Among the 7,907 predicted genes, 7,859 (99.39%) genes could be annotated by BLASTN (*E*-value < 1e^–5^) using NCBI Nr databases based on sequence homology. It should be noted that among these genes assigned to Nr database, the top three species of matched genes number are *R. toruloides* (6,180, 78.69%), followed by *R. glutinis* (1,087, 13.84%) and *Microbotryum violaceum* (226, 2.88%). In addition, 4,602 (58.20%), 4,963 (62.77%), and 7,862 (99.43%) genes could be annotated according to the KOG (Figure 1), SwissProt, and TrEMBL databases, respectively. Furthermore, 2,288 genes could be classified into three GO categories: cellular component, biological process, and molecular function (Figure 2A). To gain further insights into the gene function of *R. kratochvilovae* strain YM25235, we analyzed 3,143 genes and plotted the KEGG annotation classification statistics (Figure 2B). Summary for number of genes annotated in each database is shown in Supplementary Table 2. In addition, a repeat sequence of 557,107 bp was obtained, accounting for 2.35% of all sequences (Table 4).

**TABLE 4:** Statistics of repeat sequence classification results.

Type	Number	Length (bp)	Percentage (%)
ClassI	430	453,925	1.91
ClassI/DIRS	2	12,801	0.05
ClassI/LINE	131	66,539	0.28
ClassI/LTR/Copia	144	138,439	0.58
ClassI/LTR/Gypsy	136	229,148	0.97
ClassI/PLE| LARD	9	3,072	0.01
ClassI/TRIM	8	8,355	0.04
ClassII	47	8,908	0.04
ClassII/MITE	39	8,502	0.04
ClassII/TIR	7	395	0.00
ClassII/Unknown	1	11	0.00
PotentialHostGene	6	2,805	0.01
Unknown	315	92,433	0.39
Total	483	557,107	2.35

### Summary of Transcriptome Sequencing Data

After filtering the raw data, a total of 32.34 and 31.12 million clean reads were obtained for the treatment and control groups, respectively. The percentage of reads mapping to the *R. kratochvilovae* strain YM25235 genome was 81.1% in the treatment group and 75.9% in the control group. Uniform alignment rates across samples indicate the comparability of data between the samples (Table 5). The number of predicted novel genes was 40, and a total of 8,048 expressed genes were detected, including 8,008 known genes and 40 predicted novel genes. A total of 5,227 novel transcripts were detected, of which 4,840 belonged to the novel alternatively spliced isoforms of known protein-coding genes, 40 transcripts of novel protein-coding genes, and the remaining 347 to long non-coding RNAs. For the functional annotation, the reference gene set was aligned with sequences from major databases, including Swiss-Prot, Nr, GO, and KEGG. We performed principal component analysis (PCA) among all the samples (Supplementary Figure 1).

**TABLE 5:** Statistics of reads mapped to the genome of six samples.

Sample	Total CleanReads	Total MappingRatio (%)	Uniquely MappingRatio (%)
15-1	29,677,882	81.15	65.05
15-2	32,297,704	80.80	64.29
15-3	35,027,652	81.49	64.47
30-1	27,093,510	68.71	55.08
30-2	33,998,322	79.80	63.60
30-3	32,269,496	79.19	63.11

### Identification of Differential Gene Expression

**FIGURE 3:**
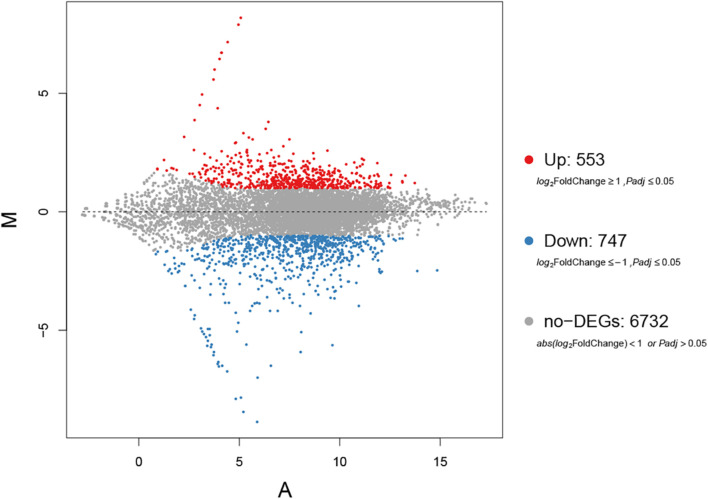
A volcano plot of differentially expressed genes (DEGs). The red spots indicate significant upregulation, whereas blue spots indicate significant downregulation. The black spots signify no significantly changed genes.

**FIGURE 4:**
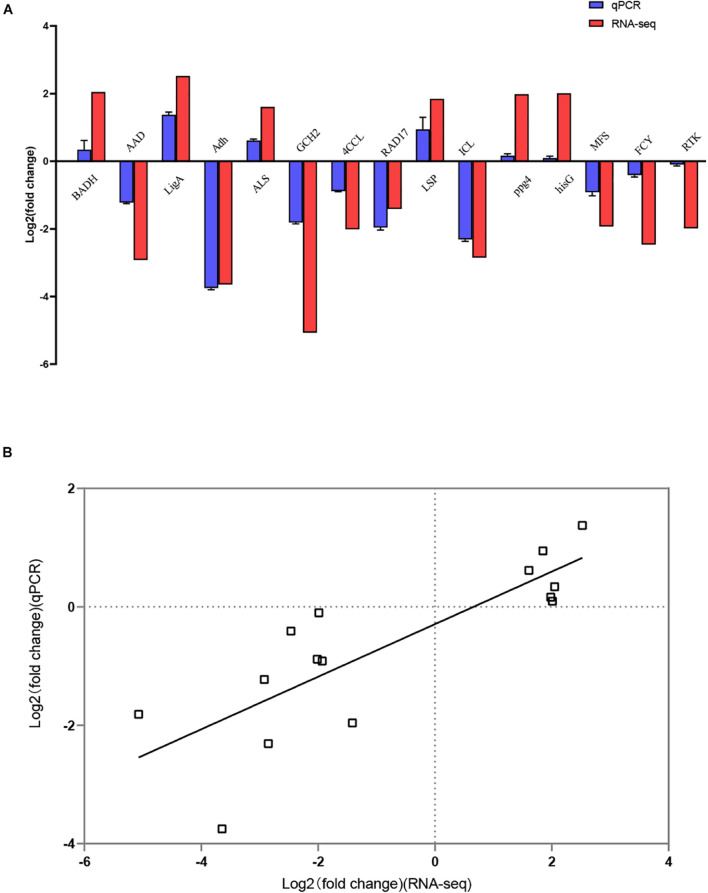
Confirmation of RNA-seq results by quantitative real-time PCR (qPCR). **(A)** Comparison of the fold change in expression of 15 selected DEGs as detected using RNA-seq and qPCR. **(B)** Correlation of the fold change in the expression of 15 DEGs analyzed by RNA-seq and qPCR.

Overall, 1,300 genes were significantly determined to be DEGs, which comprised 553 upregulated and 747 downregulated DEGs (Figure 3). They occupied 16.19% of all genes that were identified in this study. The top 10 DEGs with the most dramatic expression changes were listed in Table 6. The top three most upregulated DEGs were histone-binding protein RBBP4, molecular chaperone GrpE, protein of pyridoxal phosphate phosphatase-related family (*PLPP*). The three most downregulated DEGs were NADH oxidase (*Nox*), glycosyltransferase family 31 protein (*GT31*), and esterase/lipase (*LIP*). In addition, a minority of DEGs were annotated to hypothetical proteins, indicating their unknown functions. The qPCR analysis for all 15 DEGs represents a close correlation (Pearson’s correlation coefficients = 0.815, *p* < 0.01) in fold changes for DEGs between RNA-seq and qPCR (Figure 4), suggesting the accuracy of RNA-seq and DEG analyses.

**TABLE 6:** List of top 10 most upregulated and downregulated DEGs.

GeneID	log_2_FoldChange (Treatment/Control)	*P*_*adj*_	Gene description	Gene symbol
**The 10 most upregulated genes**				

EVM0004254	8.1856	6.22E-51	Histone-binding protein RBBP4	*RBBP4*
EVM0001626	7.9172	6.52E-46	Molecular chaperone GrpE	*GrpE*
EVM0005149	7.1616	1.97E-35	Protein of pyridoxal phosphate phosphatase-related family	*PLPP*
EVM0003618	6.7268	5.77E-30	Transcription factor	*TF*
EVM0000292	6.7266	4.83E-30	GDP-fucose protein O-fucosyltransferase family protein	*POFUT1*
EVM0002391	6.4524	1.45E-26	Nucleus export protein Brr6	*Brr6*
EVM0004860	6.0137	6.14E-22	Hypothetical protein	*/*
EVM0004609	5.5868	1.35E-17	Arylamine *N*-acetyltransferase 1	*NAT1*
EVM0001661	4.9611	1.99E-13	GATA-binding transcription factor	*GATA*
EVM0003124	4.5093	1.71E-10	Methyltransferase type 11	*MCAG_03950*

**The 10 most down-regulated genes**				

EVM0008135	–8.8632	2.02E-77	NADH oxidase	*Nox*
EVM0004703	–8.4488	1.77E-55	Glycosyltransferase family 31 protein	*GT31*
EVM0004035	–7.8793	3.70E-46	Esterase/lipase	*LIP*
EVM0004698	–7.8304	4.84E-50	Hypothetical protein	*/*
EVM0001923	–7.0013	6.58E-27	Transcription regulator HTH	*HTH*
EVM0002712	–6.7289	2.10E-28	Phenol 2-monooxygenase	*pheA*
EVM0007841	–6.5184	1.87E-27	Putative Nucleoside-diphosphate-sugar epimerase	*ytbQ*
EVM0004887	–6.4840	3.33E-101	Pyridoxine biosynthesis protein	*PDX1*
EVM0004965	–6.4789	3.67E-26	Zinc finger, RING-type protein	*Znf_RING*
EVM0006689	–6.4149	2.88E-26	Hypothetical protein	*/*

### GO and KEGG Enrichment Analyses of DEGs

**FIGURE 5:**
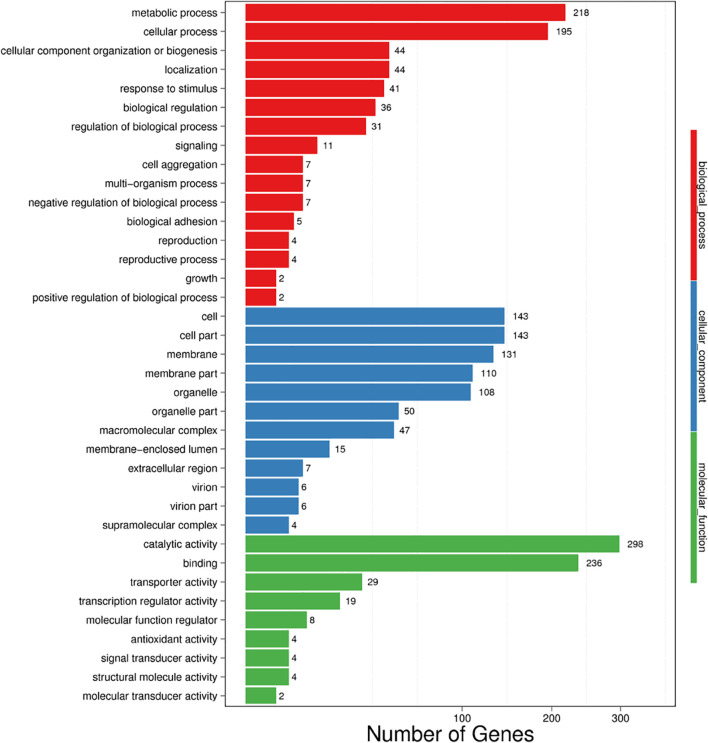
Vertical histogram presentation of DEG-enriched GO terms. BP (red): biological process, CC (blue): cellular component, and MF (green): molecular function.

All DEGs were divided into three GO categories: biological process (19 terms), molecular function (18 terms), and cellular component (6 terms) (Figure 5). Among the BP terms, the three GO terms containing the highest number of DEGs were metabolic process, cellular process, and cellular component organization or biogenesis. Moreover, some BP terms involving response to low temperature were detected, such as biological process, negative regulation of biological process and growth. In the MF category, catalytic activity, binding, and transporter activity were the top three terms, and for the CC category, cell, cell part, and membrane were the top three terms. In addition, the membrane part ranked fourth.

**FIGURE 6:**
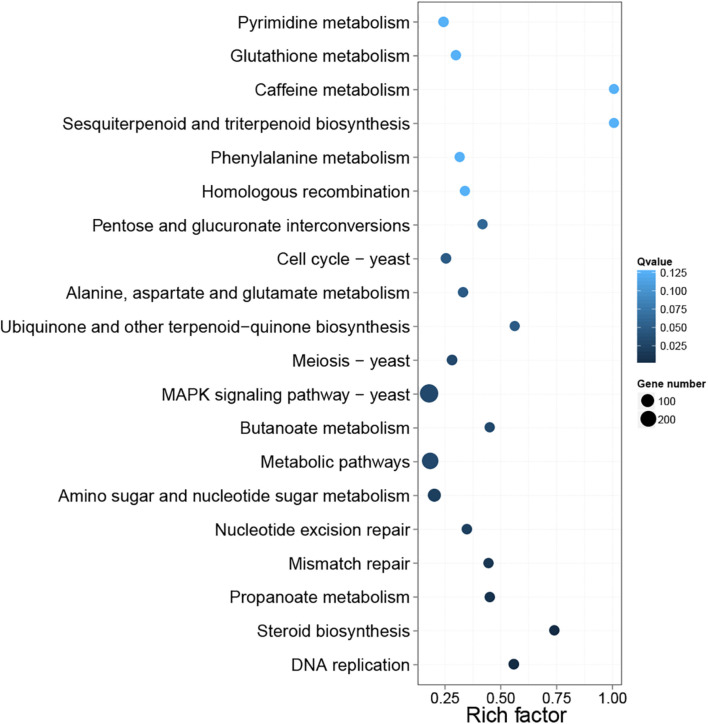
Top 20 differential gene pathway enrichment results. The X-axis represents the enrichment factor value, and the Y-axis represents the pathway name. Color represents the *Q* value, with smaller values representing more significant enrichment results. The size of the dots represents the number of DEGs. Rich factor refers to the enrichment factor value, and a larger value indicates a more pronounced enrichment result.

In the pathway enrichment analysis, 107 KEGG pathways were enriched by DEGs (Supplementary Table 3). Furthermore, the top 20 KEGG pathways were enriched by DEGs and are presented in Figure 6. MAPK signaling pathway-yeast (ko04011) and metabolic pathways (ko01100) contained the most DEGs. The remaining KEGG terms were primarily associated with amino acid metabolism [e.g., alanine, aspartate, and glutamate metabolism (ko00250), valine, leucine, and isoleucine degradation (ko00310), and lysine degradation (ko00310)], carbohydrate metabolism [e.g., amino sugar and nucleotide sugar metabolism (ko00520) and propanoate metabolism (ko00640)], lipid metabolism [i.e., steroid biosynthesis (ko00100)], cell growth and death (i.e., cell cycle-yeast [ko04111] and meiosis yeast (ko04113), and translation [i.e., RNA transport (ko03013) and mRNA surveillance pathway (ko03015)]. In addition, all 1,300 DEGs included in different pathways were listed in Supplementary File 2 List of DEGs.

## Discussion

Compared with several *Rhodosporidium* strains that have been sequenced, *R. kratochvilovae* strain YM25235 was isolated from Chenghai Lake in Yunnan, China. This plateau lake locates at the border of Tibet Plateau and Yunnan plateau, where the water temperature ranges from 2 to 31°C, with an average temperature of about 15°C (Wan et al., 2005). The high-quality genomic and transcriptomic data of *R. kratochvilovae* strain YM25235 were obtained and the molecular mechanism regarding low temperature response of *R. kratochvilovae* strain YM25235 was explored in this study. The comparative transcriptomic analysis of *R. kratochvilovae* strain YM25235 treated by low temperature and control temperature identified several DEGs primarily involved in BP functions (e.g., metabolism, regulation processes, and stress response). It was established that low temperature induced changes in the biological process of *R. kratochvilovae* strain YM25235. In addition, the terms associated with metabolism were overrepresented (77, 71.96% of all terms) among the enriched KEGG pathways and adjustment of metabolism function due to gene expression changes primarily contributed to the low temperature response of *R. kratochvilovae* strain YM25235.

In particular, steroids can serve as important components of cell membranes, which can alter the fluidity of cell membranes (Fernández-Cabezón et al., 2018). Moreover, triterpenoid serves as a precursor for steroid biosynthesis (Davis and Croteau, 2000). In this study, several KEGG pathways involved in sesquiterpenoid and triterpenoid biosynthesis were significantly enriched. This evidence suggested that steroids could act as a crucial factor for changes in the protective ability of *R. kratochvilovae* strain YM25235 against low temperature by altering the fluidity of cell membranes. Therefore, genes involved in steroid products are useful targets in genetic manipulation to improve the cold resistance of *R. kratochvilovae* strain YM25235. Another analysis revealed that the DEGs of the biosynthesis of steroid pathway were overwhelmingly upregulated (Supplementary Figure 2), further supporting that *R. kratochvilovae* strain YM25235 adapts to the low temperature environment by adjusting the biosynthesis of steroids. Ubiquinone and other terpenoid quinone are involved in MVA (Dallner and Sindelar, 2000). The MVA pathway, in turn, is upstream of not only steroid biosynthesis but also carotenoid biosynthesis (Katsuki and Bloch, 1967; Groeneveld, 1999). However, carotenoids could modulate membrane fluidity because carotenoids can stabilize the membrane to respond to low temperature (Chattopadhyay et al., 1997; Jagannadham et al., 2000). Low temperature stress also leads to increased intracellular reactive oxygen species (ROS) (Gasch, 2003), more carotenoids biosynthesis can mitigate cellular damage caused by ROS (Breierová et al., 2018). In this study, ubiquinone and other terpenoid quinone biosynthesis were significantly enriched. Certain amino acids, including alanine, aspartate, glutamate, and phenylalanine metabolic pathways were enriched, suggesting an altered utilization of amino acids by *R. kratochvilovae* strain YM25235 at low temperature, similar to the findings reported by Beltran et al. (2007). In addition, glutathione metabolism was significantly enriched, glutathione (GSH) protects the cells by neutralizing (i.e., reducing) ROS (Wu et al., 2004), Thus, GSH might protect *R. kratochvilovae* strain YM25235 from increased ROS at low temperature. In addition, the DNA replication, mismatch repair, and nucleotide excision repair pathways were enriched in the current study. Previous studies have reported genes whose expression is affected by low temperature associated with DNA replication (Driessen et al., 2014). Thus, it can be inferred that the changes occurring in the levels of DNA replication might be involved in the important biological processes in *R. kratochvilovae* strain YM25235 acting at low temperature. Moreover, most of the downregulated DEGs were related to propanoate metabolism, valine, leucine, and isoleucine degradation, pentose, and glucuronate interconversion. We speculate that low temperature limits the progression of these biological responses (Supplementary Figure 2).

MAPKs are serine/threonine kinases that, when phosphorylated, enter the nucleus and phosphorylate several transcription factors, enzymes, and other proteins that modulate the cellular activity (Cowan and Storey, 2003). The MAPK gene family is well known for its function in transmitting stress signals from the environment to the cell nucleus (Cowan and Storey, 2003). Significant enrichment of the MAPK pathway in this study indicated the implication of MAPKs in transmitting stress signals from the environment to the cell nucleus in *R. kratochvilovae* strain YM25235 at low temperature. Traditionally, the HOG pathway, a conserved MAPK cascade, has been considered a specific signaling pathway, responding to changes in external solute concentrations (Hohmann, 2002; Westfall et al., 2004). However, the HOG pathway was demonstrated to function as a transducer of cold stimuli (Panadero et al., 2006). *Hog1*, as a key gene of the HOG pathway was implicated in response to low temperature stress in our lab study (unpublished). However, *Hog1* was not identified as a DEG in this study. We speculate that this process is achieved at the protein level or by certain other mechanisms and is not manifested by differences in the transcript levels.

The top 10 identified DEGs might play a key role for *R. kratochvilovae* strain YM25235 when grown at low temperature. The *RBBP4* gene has been reported to regulate signaling through the MAPK pathway (Li et al., 2019). The activity of GrpE, a nucleotide exchange factor for the heat shock protein DnaK, is downregulated in response to increasing temperature (Bracher and Verghese, 2015). In this study, the expression of *GrpE* significantly increased at low temperature, indicating that *GrpE* is a key molecule participating in the response of *R. kratochvilovae* strain YM25235 to low temperature. The nucleus export protein Brr6 is involved in mRNA and protein export from the nucleus and contribute to mitosis (Lo Presti et al., 2007). The translational activity of mRNA-based protein biosynthesis greatly decreased at low temperatures (Jones and Inouye, 1996). Vitamin B6 as oxygen reactive species scavengers and factors was able to increase resistance to biotic and abiotic stress (Ehrenshaft et al., 1999; Bilski et al., 2000). This vitamin comprises six interconvertible pyridine compounds (vitamers) such as pyridoxal 5’-phosphate (PLP) and five others (ShuoHao et al., 2019). Gene that codes a protein of pyridoxal phosphate phosphatase-related family (PLPP) were significantly upregulated in this study, indicating that *PLPP* plays a key role in responding to low temperature. In this study, the transcript levels of certain genes involved in regulating the transcription were significantly changed at low temperature. *HTH* and *GATA* were detected with significant changes at the transcript level. Most of these proteins were transcription activators and are known to negatively regulate their expression (Baumeister et al., 1992). Thus, the results revealed potential key roles of these genes for *R. kratochvilovae* strain YM25235 to grow at low temperature. Notably, the functions of several top DEGs remain largely unexplored, and they cannot be linked well to the response of *R. kratochvilovae* strain YM25235 to low temperature. However, this study is the first to reveal their acute response of *R. kratochvilovae* strain YM25235 to low temperature.

Membrane rigidity increases when cells are incubated at low temperatures *in vitro* (Hayashi and Maeda, 2006). Microorganisms overcome the membrane stiffness at low temperatures by adapting to the environment to keep the membrane fluidity constant to a degree (Morgan-Kiss et al., 2006). To accomplish this, cold stress triggers a coordinated response that induces fatty acid desaturases and dehydrogenases, which in turn increase the ratio of polyunsaturated to saturated fatty acids and/or actually decrease the length of fatty acid chains in the membrane (Dickens and Thompson, 1981; Avery et al., 1995; Carty et al., 1999). The PUFAs association with low temperature response in *R. kratochvilovae* strain YM25235 has been confirmed in our previous study (Cui et al., 2016). However, no pathway and no DEGs related to PUFA biosynthesis were significantly enriched in this study. This is because the response to low temperature stress is a complex process. We speculate that this process may be achieved at the protein level or by certain other mechanisms and is not manifested by differences in the transcript levels.

## Conclusion

In this study, the genome-wide transcriptional changes of *R. kratochvilovae* strain YM25235 at low temperature was obtained by whole genome sequencing of *R. kratochvilovae* strain YM25235 and transcriptome sequencing from low temperature (15°C) and normal temperature (30°C) groups. Likewise, this study also showed that there exist genes whose functions are still not known and certain mechanisms of response to low temperature cannot be elucidated. Further experimental verifications are needed. Notably, despite the DEGs obtained in this study are related to low temperature response, further verification for DEGs directly involved in the response is required in the future.

## Data Availability Statement

The datasets presented in this study can be found in online repositories. The names of the repository/repositories and accession number(s) can be found below: https://www.ncbi.nlm.nih.gov/, PRJNA739038 (genome sequencing and assembly) and PRJNA739250 (Transcriptome).

## Author Contributions

RG, QZ, and Q-LZ contributed to the conception and design of the study. RG and XZ did the laboratory work and acquired all raw data. RG and MH did the bioinformatic analyses. YW and XJ provided the research materials. RG wrote the first draft of the manuscript. QZ and Q-LZ wrote sections of the manuscript. All authors contributed to manuscript revision, read, and approved the submitted version.

## Conflict of Interest

The authors declare that the research was conducted in the absence of any commercial or financial relationships that could be construed as a potential conflict of interest.

## Publisher’s Note

All claims expressed in this article are solely those of the authors and do not necessarily represent those of their affiliated organizations, or those of the publisher, the editors and the reviewers. Any product that may be evaluated in this article, or claim that may be made by its manufacturer, is not guaranteed or endorsed by the publisher.

## References

[B1] Ageitos J. M. Vallejo J. A. Veiga-Crespo P. Villa T. G. (2011). Oily yeasts as oleaginous cell factories. *Appl. Microbiol. Biotechnol.* 90 1219–1227. 10.1007/s00253-011-3200-z 21465305

[B2] Aguilera J. Randez-Gil F. Prieto J. A. (2007). Cold response in *Saccharomyces cerevisiae*: new functions for old mechanisms. *FEMS Microbiol. Rev.* 31 327–341. 10.1111/j.1574-6976.2007.00066.x 17298585

[B3] Alioto T. Blanco E. Parra G. Guigó R. (2018). Using geneid to Identify Genes. *Curr. Protoc. Bioinform.* 64:e56. 10.1002/cpbi.56 30332532

[B4] Ashburner M. Ball C. A. Blake J. A. Botstein D. Butler H. Cherry J. M. (2000). Gene ontology: tool for the unification of biology. *Nat. Genet.* 25 25–29. 10.1038/75556 10802651 PMC3037419

[B5] Avery S. V. Lloyd D. Harwood J. L. (1995). Temperature-dependent changes in plasma-membrane lipid order and the phagocytotic activity of the amoeba *Acanthamoeba castellanii* are closely correlated. *Biochem. J.* 312 811–816. 10.1042/bj3120811 8554525 PMC1136187

[B6] Baumeister R. Müller G. Hecht B. Hillen W. (1992). Functional roles of amino acid residues involved in forming the alpha-helix-turn-alpha-helix operator DNA binding motif of Tet repressor from Tn10. *Proteins* 14 168–177. 10.1002/prot.340140204 1409566

[B7] Beltran G. Rozès N. Mas A. Guillamón J. M. (2007). Effect of low-temperature fermentation on yeast nitrogen metabolism. *World J. Microbiol. Biotechnol.* 23 809–815. 10.1007/s11274-006-9302-6

[B8] Bilski P. Li M. Ehrenshaft M. Daub M. Chignell C. (2000). Vitamin B6 (pyridoxine) and its derivatives are efficient singlet oxygen quenchers and potential fungal antioxidants. *Photochem. Photobiol.* 71 129–134. 10.1562/0031-865520000710129SIPVBP2.0.CO2 10687384

[B9] Boeckmann B. Bairoch A. Apweiler R. Blatter M.-C. Estreicher A. Gasteiger E. (2003). The SWISS-PROT protein knowledgebase and its supplement TrEMBL in 2003. *Nucleic Acids Res.* 31 365–370. 10.1093/nar/gkg095 12520024 PMC165542

[B10] Bracher A. Verghese J. (2015). The nucleotide exchange factors of Hsp70 molecular chaperones. *Front. Mol. Biosci.* 2:10. 10.3389/fmolb.2015.00010 26913285 PMC4753570

[B11] Breierová E. Čertík M. Márová I. Vadkertiová R. (2018). The effect of Zn(II) ions and reactive oxygen on the uptake of zinc and production of carotenoids by selected red yeasts. *Chem. Biodiv.* 15:e1800069. 10.1002/cbdv.201800069 29655310

[B12] Burge C. Karlin S. (1997). Prediction of complete gene structures in human genomic DNA. *J. Mol. Biol.* 268 78–94. 10.1006/jmbi.1997.0951 9149143

[B13] Buzzini P. Innocenti M. Turchetti B. Libkind D. van Broock M. Mulinacci N. (2007). Carotenoid profiles of yeasts belonging to the genera *Rhodotorula, Rhodosporidium, Sporobolomyces*, and *Sporidiobolus*. *Can. J. Microbiol.* 53 1024–1031. 10.1139/W07-068 17898860

[B14] Calvo A. M. Gardner H. W. Keller N. P. (2001). Genetic connection between fatty acid metabolism and sporulation in *Aspergillus nidulans*. *J. Biol. Chem.* 276 25766–25774. 10.1074/jbc.M100732200 11352908

[B15] Campbell M. A. Haas B. J. Hamilton J. P. Mount S. M. Buell C. R. (2006). Comprehensive analysis of alternative splicing in rice and comparative analyses with Arabidopsis. *BMC Genom.* 7:327. 10.1186/1471-2164-7-327 17194304 PMC1769492

[B16] Carrasco M. Alcaíno J. Cifuentes V. Baeza M. (2017). Purification and characterization of a novel cold adapted fungal glucoamylase. *Microbial. Cell Factories* 16:75. 10.1186/s12934-017-0693-x 28464820 PMC5414198

[B17] Carty S. M. Sreekumar K. R. Raetz C. R. H. (1999). Effect of cold shock on lipid a biosynthesis in *Escherichia coli*. *J. Biol. Chem.* 274 9677–9685. 10.1074/jbc.274.14.9677 10092655

[B18] Castrillo M. Luque E. M. Pardo-Medina J. Limón M. C. Corrochano L. M. Avalos J. (2018). Transcriptional basis of enhanced photoinduction of carotenoid biosynthesis at low temperature in the fungus *Neurospora crassa*. *Res. Microbiol.* 169 78–89. 10.1016/j.resmic.2017.11.003 29203212

[B19] Chattopadhyay M. Jagannadham M. Vairamani M. Shivaji S. (1997). Carotenoid pigments of an Antarctic psychrotrophic bacterium *Micrococcus roseus*: temperature dependent biosynthesis, structure, and interaction with synthetic membranes. *Biochem. Biophys. Res. Commun.* 239 85–90. 10.1006/bbrc.1997.7433 9345274

[B20] Chen N. (2004). Using repeat masker to identify repetitive elements in genomic sequences. *Curr. Protoc. Bioinform.* Chapter 4:Unit4.10. 10.1002/0471250953.bi0410s05 18428725

[B21] Clarke S. D. (2001). Polyunsaturated fatty acid regulation of gene transcription: a molecular mechanism to improve the metabolic syndrome. *J. Nut.* 131 1129–1132. 10.1093/jn/131.4.1129 11285313

[B22] Cock P. J. Fields C. J. Goto N. Heuer M. L. Rice P. M. (2010). The sanger FASTQ file format for sequences with quality scores, and the Solexa/Illumina FASTQ variants. *Nucleic Acids Res.* 38 1767–1771. 10.1093/nar/gkp1137 20015970 PMC2847217

[B23] Conesa A. Gotz S. Garcia-Gomez J. M. Terol J. Talon M. Robles M. (2005). Blast2GO: a universal tool for annotation, visualization and analysis in functional genomics research. *Bioinformatics* 21 3674–3676. 10.1093/bioinformatics/bti610 16081474

[B24] Cowan K. J. Storey K. B. (2003). Mitogen-activated protein kinases: new signaling pathways functioning in cellular responses to environmental stress. *J. Exp. Biol.* 206 1107–1115. 10.1242/jeb.00220 12604570

[B25] Cui J. He S. Ji X. Lin L. Wei Y. Zhang Q. (2016). Identification and characterization of a novel bifunctional Δ12/Δ15-fatty acid desaturase gene from Rhodosporidium kratochvilovae. *Biotechnol. Lett.* 38 1155–1164. 10.1007/s10529-016-2090-7 27032802

[B26] Dallner G. Sindelar P. J. (2000). Regulation of ubiquinone metabolism. *Free Radical Biol. Med.* 29 285–294. 10.1016/s0891-5849(00)00307-5 11035257

[B27] Davis E. M. Croteau R. (2000). “Cyclization Enzymes in the Biosynthesis of Monoterpenes, Sesquiterpenes, and Diterpenes,” in *Biosynthesis: Aromatic Polyketides, Isoprenoids, Alkaloids*, eds Leeper F. J. Vederas J. C. (Berlin: Springer Berlin Heidelberg), 53–95. 10.1007/3-540-48146-X_2

[B28] Deng Y. Y. Li J. Q. Wu S. F. Zhu Y. Chen Y. W. He F. C. (2006). Integrated nr database in protein annotation system and its localization. *Computer Eng.* 32 71–72.

[B29] Dickens B. F. Thompson G. A. (1981). Rapid membrane response during low-temperature acclimation Correlation of early changes in the physical properties and lipid composition of *Tetrahymena microsomal* membranes. *Biochim. et Biophys. Acta (BBA) Biomembranes* 644 211–218. 10.1016/0005-2736(81)90377-1 6789874

[B30] Dinh H. V. Suthers P. F. Chan S. H. J. Shen Y. Xiao T. Deewan A. (2019). A comprehensive genome-scale model for *Rhodosporidium toruloides* IFO0880 accounting for functional genomics and phenotypic data. *Metabolic Eng. Commun.* 9:e00101. 10.1016/j.mec.2019.e00101 31720216 PMC6838544

[B31] Driessen R. P. C. Sitters G. Laurens N. Moolenaar G. F. Wuite G. J. L. Goosen N. (2014). Effect of temperature on the intrinsic flexibility of DNA and its interaction with architectural proteins. *Biochemistry* 53 6430–6438. 10.1021/bi500344j 25291500 PMC5451147

[B32] Eddy S. R. (1998). Profile hidden markov models. *Bioinformatics* 14 755–763. 10.1093/bioinformatics/14.9.755 9918945

[B33] Edgar R. C. Myers E. W. (2005). PILER: identification and classification of genomic repeats. *Bioinformatics* 21(Suppl. 1) i152–i158. 10.1093/bioinformatics/bti1003 15961452

[B34] Ehrenshaft M. Bilski P. Li M. Y. Chignell C. F. Daub M. E. (1999). A highly conserved sequence is a novel gene involved in de novo vitamin B6 biosynthesis. *Proc. Natl. Acad. Sci. U.S.A.* 96 9374–9378. 10.1073/pnas.96.16.9374 10430950 PMC17790

[B35] Fernández-Cabezón L. Galán B. García J. L. (2018). New insights on steroid biotechnology. *Front. Microbiol.* 9:958. 10.3389/fmicb.2018.00958 29867863 PMC5962712

[B36] Finn R. D. Coggill P. Eberhardt R. Y. Eddy S. R. Mistry J. Mitchell A. L. (2016). The Pfam protein families database: towards a more sustainable future. *Nucleic Acids Res.* 44 D279–D285. 10.1093/nar/gkv1344 26673716 PMC4702930

[B37] Firdaus-Raih M. Hashim N. H. F. Bharudin I. Abu Bakar M. F. Huang K. K. Alias H. (2018). The *Glaciozyma antarctica* genome reveals an array of systems that provide sustained responses towards temperature variations in a persistently cold habitat. *PLoS One* 13:e0189947. 10.1371/journal.pone.0189947 29385175 PMC5791967

[B38] García-Ríos E. Morard M. Parts L. Liti G. Guillamón J. M. (2017). The genetic architecture of low-temperature adaptation in the wine yeast *Saccharomyces cerevisiae*. *BMC Genom.* 18:159. 10.1186/s12864-017-3572-2 28196526 PMC5310122

[B39] Gasch A. P. (2003). “The environmental stress response: a common yeast response to diverse environmental stresses,” in *Yeast Stress Responses*, eds Hohmann S. Mager W. H. (Berlin: Springer Berlin Heidelberg), 11–70. 10.1007/3-540-45611-2_2

[B40] Groeneveld H. W. (1999). Tracing steroid synthesis in plants. *Crit. Rev. Biochem. Mol. Biol.* 34 59–69. 10.1080/10409239991209192 10333384

[B41] Haas B. J. Salzberg S. L. Zhu W. Pertea M. Allen J. E. Orvis J. (2008). Automated eukaryotic gene structure annotation using evidencemodeler and the program to assemble spliced alignments. *Genome Biol.* 9:R7. 10.1186/gb-2008-9-1-r7 18190707 PMC2395244

[B42] Han Y. Wessler S. R. (2010). MITE-hunter: a program for discovering miniature inverted-repeat transposable elements from genomic sequences. *Nucleic Acids Res.* 38:e199. 10.1093/nar/gkq862 20880995 PMC3001096

[B43] Hayashi M. Maeda T. (2006). Activation of the HOG pathway upon cold stress in *Saccharomyces cerevisiae*. *J. Biochem.* 139 797–803. 10.1093/jb/mvj089 16672281

[B44] He J. Cui Z. Ji X. Luo Y. Wei Y. Zhang Q. (2019). Novel histidine kinase gene HisK2301from *Rhodosporidium kratochvilovae* contributes to cold adaption by promoting biosynthesis of polyunsaturated fatty acids and glycerol. *J. Agric. Food Chem.* 67 653–660. 10.1021/acs.jafc.8b04859 30558417

[B45] Hohmann S. (2002). Osmotic stress signaling and osmoadaptation in yeasts. *Microbiol. Mol. Biol. Rev. MMBR* 66 300–372. 10.1128/MMBR.66.2.300-372.2002 12040128 PMC120784

[B46] Hu J. Ji L. (2016). Draft genome sequences of *Rhodosporidium toruloides* strains ATCC 10788 and ATCC 10657 with compatible mating types. *Genome Announ.* 4:e00098-16. 10.1128/genomeA.00098-16 26966203 PMC4786659

[B47] Jagannadham M. V. Chattopadhyay M. K. Subbalakshmi C. Vairamani M. Narayanan K. Mohan Rao C. (2000). Carotenoids of an Antarctic psychrotolerant bacterium, Sphingobacterium antarcticus, and a mesophilic bacterium, *Sphingobacterium multivorum*. *Arch. Microbiol.* 173 418–424. 10.1007/s002030000163 10896223

[B48] Jagtap S. S. Bedekar A. A. Liu J.-J. Jin Y.-S. Rao C. V. (2019). Production of galactitol from galactose by the oleaginous yeast *Rhodosporidium toruloides* IFO0880. *Biotechnol. Biofuels* 12:250. 10.1186/s13068-019-1586-5 31636709 PMC6798376

[B49] Jagtap S. S. Rao C. V. (2018). Production of d-arabitol from d-xylose by the oleaginous yeast *Rhodosporidium toruloides* IFO0880. *Appl. Microbiol. Biotechnol.* 102 143–151. 10.1007/s00253-017-8581-1 29127468

[B50] Jantzen S. G. Sutherland B. J. Minkley D. R. Koop B. F. (2011). GO trimming: systematically reducing redundancy in large gene ontology datasets. *BMC Res. Notes* 4:267. 10.1186/1756-0500-4-267 21798041 PMC3160396

[B51] Jiang W. Lv Y. Cheng L. Yang K. Bian C. Wang X. (2019). Whole-genome sequencing of the giant devil catfish, *Bagarius yarrelli*. *Genome Biol. Evol.* 11 2071–2077. 10.1093/gbe/evz143 31274158 PMC6681832

[B52] Jones P. G. Inouye M. (1996). RbfA, a 30S ribosomal binding factor, is a cold-shock protein whose absence triggers the cold-shock response. *Mol. Microbiol.* 21 1207–1218. 10.1111/j.1365-2958.1996.tb02582.x 8898389

[B53] Jurka J. Kapitonov V. V. Pavlicek A. Klonowski P. Kohany O. Walichiewicz J. (2005). Repbase update, a database of eukaryotic repetitive elements. *Cytogenet Genome Res.* 110 462–467. 10.1159/000084979 16093699

[B54] Kanehisa M. Goto S. Kawashima S. Okuno Y. Hattori M. (2004). The KEGG resource for deciphering the genome. *Nucleic Acids Res.* 32 D277–D280. 10.1093/nar/gkh063 14681412 PMC308797

[B55] Katsuki H. Bloch K. (1967). Studies on the biosynthesis of ergosterol in yeast: formation of methylated intermediates. *J. Biol. Chem.* 242 222–227. 10.1016/S0021-9258(19)81452-7 6016607

[B56] Keilwagen J. Wenk M. Erickson J. L. Schattat M. H. Grau J. Hartung F. (2016). Using intron position conservation for homology-based gene prediction. *Nucleic Acids Res.* 44:e89. 10.1093/nar/gkw092 26893356 PMC4872089

[B57] Kim D. Langmead B. Salzberg S. L. (2015). HISAT: a fast spliced aligner with low memory requirements. *Nat. Methods* 12 357–360. 10.1038/nmeth.3317 25751142 PMC4655817

[B58] Koren S. Walenz B. P. Berlin K. Miller J. R. Bergman N. H. Phillippy A. M. (2017). Canu: scalable and accurate long-read assembly via adaptive k-mer weighting and repeat separation. *Genome Res.* 27 722–736. 10.1101/gr.215087.116 28298431 PMC5411767

[B59] Korf I. (2004). Gene finding in novel genomes. *BMC Bioinform.* 5:59. 10.1186/1471-2105-5-59 15144565 PMC421630

[B60] Langmead B. Salzberg S. L. (2012). Fast gapped-read alignment with bowtie 2. *Nat. Methods* 9 357–359. 10.1038/nmeth.1923 22388286 PMC3322381

[B61] Li B. Dewey C. N. (2011). RSEM: accurate transcript quantification from RNA-Seq data with or without a reference genome. *BMC Bioinform.* 12:323. 10.1186/1471-2105-12-323 21816040 PMC3163565

[B62] Li Y. D. Lv Z. Xie H. Y. Zheng S. S. (2019). Retinoblastoma binding protein 4 up-regulation is correlated with hepatic metastasis and poor prognosis in colon cancer patients. *Hepatobil. Pancreat. Dis. Int.* 18 446–451. 10.1016/j.hbpd.2019.08.006 31501018

[B63] Livak K. J. Schmittgen T. D. (2001). Analysis of relative gene expression data using real-time quantitative PCR and the 2−ΔΔCT method. *Methods* 25 402–408. 10.1006/meth.2001.1262 11846609

[B64] Lo Presti L. Cockell M. Cerutti L. Simanis V. Hauser P. M. (2007). Functional characterization of Pneumocystis carinii brl1 by transspecies complementation analysis. *Eukaryot Cell* 6 2448–2452. 10.1128/EC.00321-07 17993570 PMC2168235

[B65] Long Y. Yan J. Song G. Li X. Li X. Li Q. (2015). Transcriptional events co-regulated by hypoxia and cold stresses in *Zebrafish larvae*. *BMC Genomics* 16:385. 10.1186/s12864-015-1560-y 25975375 PMC4432979

[B66] Love M. I. Huber W. Anders S. (2014). Moderated estimation of fold change and dispersion for RNA-seq data with DESeq2. *Genome Biol.* 15:550. 10.1186/s13059-014-0550-8 25516281 PMC4302049

[B67] MacDonald M. C. Arivalagan P. Barre D. E. MacInnis J. A. D’Cunha G. B. (2016). *Rhodotorula glutinis* Phenylalanine/tyrosine ammonia lyase enzyme catalyzed synthesis of the methyl ester of para-hydroxycinnamic acid and its potential antibacterial activity. *Front. Microbiol.* 7:281. 10.3389/fmicb.2016.00281 27014206 PMC4781862

[B68] Majoros W. H. Pertea M. Salzberg S. L. (2004). Tigr scan and glimmer HMM: two open source ab initio eukaryotic gene-finders. *Bioinformatics* 20 2878–2879. 10.1093/bioinformatics/bth315 15145805

[B69] Margesin R. Miteva V. (2011). Diversity and ecology of psychrophilic microorganisms. *Res. Microbiol.* 162 346–361. 10.1016/j.resmic.2010.12.004 21187146

[B70] Miccoli C. Palmieri D. De Curtis F. Lima G. Ianiri G. Castoria R. (2018). Complete genome sequence of the biocontrol agent yeast *Rhodotorula kratochvilovae* strain LS11. *Genome Announ.* 6:e00120-18. 10.1128/genomeA.00120-18 29519831 PMC5843719

[B71] Morgan-Kiss R. M. Priscu J. C. Pocock T. Gudynaite-Savitch L. Huner N. P. A. (2006). Adaptation and acclimation of photosynthetic microorganisms to permanently cold environments. *Microbiol. Mol. Biol. Rev. MMBR* 70 222–252. 10.1128/MMBR.70.1.222-252.2006 16524924 PMC1393254

[B72] Murata Y. Homma T. Kitagawa E. Momose Y. Sato M. S. Odani M. (2006). Genome-wide expression analysis of yeast response during exposure to 4 C. *Extremophiles* 10 117–128. 10.1007/s00792-005-0480-1 16254683

[B73] Panadero J. Pallotti C. Rodríguez-Vargas S. Randez-Gil F. Prieto J. A. (2006). A downshift in temperature activates the high osmolarity glycerol (HOG) pathway, which determines freeze tolerance in *Saccharomyces cerevisiae*^∗^. *J. Biol. Chem.* 281 4638–4645. 10.1074/jbc.M512736200 16371351

[B74] Pham K. D. Shida Y. Miyata A. Takamizawa T. Suzuki Y. Ara S. (2020). Effect of light on carotenoid and lipid production in the oleaginous yeast Rhodosporidium toruloides. *Biosci. Biotechnol. Biochem.* 84 1501–1512. 10.1080/09168451.2020.1740581 32189572

[B75] Price A. L. Jones N. C. Pevzner P. A. (2005). De novo identification of repeat families in large genomes. *Bioinformatics* 21(Suppl. 1) i351–i358. 10.1093/bioinformatics/bti1018 15961478

[B76] Ruan J. Li H. (2019). Fast and accurate long-read assembly with wtdbg2. *Nat. Methods* 17 155–158. 10.1101/530972 31819265 PMC7004874

[B77] Sampath H. Ntambi J. M. (2004). Polyunsaturated fatty acid regulation of gene expression. *Nutrit. Rev.* 62 333–339. 10.1111/j.1753-4887.2004.tb00058.x 15497766

[B78] ShuoHao H. Jing L. Jie Z. JianYun Z. LongQuan H. (2019). Identification and characterization of a pyridoxal 5’-phosphate phosphatase in tobacco plants. *Plant Sci.* 278 88–95. 10.1016/j.plantsci.2018.10.014 30471733

[B79] Simão F. Waterhouse R. M. Panagiotis I. Kriventseva E. V. Zdobnov E. M. (2015). BUSCO: assessing genome assembly and annotation completeness with single-copy orthologs. *Bioinformatics* 31, 3210–3212. 10.1093/bioinformatics/btv351 26059717

[B80] Stanke M. Waack S. (2003). Gene prediction with a hidden Markov model and a new intron submodel. *Bioinformatics* 19(Suppl. 2) ii215–ii225. 10.1093/bioinformatics/btg1080 14534192

[B81] Tatusov R. L. Galperin M. Y. Natale D. A. Koonin E. V. (2000). The COG database: a tool for genome-scale analysis of protein functions and evolution. *Nucleic Acids Res.* 28 33–36. 10.1093/nar/28.1.33 10592175 PMC102395

[B82] Tran T. N. Ngo D.-H. Nguyen N. T. Ngo D.-N. (2019). Draft genome sequence data of Rhodosporidium toruloides VN1, a strain capable of producing natural astaxanthin. *Data Brief* 26:104443. 10.1016/j.dib.2019.104443 31528677 PMC6742850

[B83] Uprety B. K. Dalli S. S. Rakshit S. K. (2017). Bioconversion of crude glycerol to microbial lipid using a robust oleaginous yeast *Rhodosporidium toruloides* ATCC 10788 capable of growing in the presence of impurities. *Energy Conv. Manag.* 135 117–128. 10.1016/j.enconman.2016.12.071

[B84] Wan G. J. Chen J. A. Wu F. C. Xu S. Q. Bai Z. G. Wan E. Y. (2005). Coupling between 210Pbex and organic matter in sediments of a nutrient-enriched lake: an example from Lake Chenghai, China. *Chem. Geol.* 224 223–236. 10.1016/j.chemgeo.2005.07.025

[B85] Wang C. Chen Y. Zhou H. Li X. Tan Z. (2020). Adaptation mechanisms of *Rhodococcus* sp. CNS16 under different temperature gradients: Physiological and transcriptome. *Chemosphere* 238:124571. 10.1016/j.chemosphere.2019.124571 31472351

[B86] Wang J. Chen W. Nian H. Ji X. Lin L. Wei Y. (2017). Inhibition of polyunsaturated fatty acids synthesis decreases growth rate and membrane fluidity of *Rhodosporidium kratochvilovae* at low temperature. *Lipids* 52 729–735. 10.1007/s11745-017-4273-y 28660529

[B87] Wang Q. Tan X. Jiao S. You F. Zhang P. J. (2014). Analyzing cold tolerance mechanism in transgenic zebrafish (Danio rerio). *PLoS One* 9:e102492. 10.1371/journal.pone.0102492 25058652 PMC4109919

[B88] Wang Y. Zhang S. Zhu Z. Shen H. Lin X. Jin X. (2018). Systems analysis of phosphate-limitation-induced lipid accumulation by the oleaginous yeast *Rhodosporidium toruloides*. *Biotechnol. Biofuels* 11:148. 10.1186/s13068-018-1134-8 29849765 PMC5968551

[B89] Westfall P. J. Ballon D. R. Thorner J. (2004). When the stress of your environment makes you go hog wild. *Science* 306:1511. 10.1126/science.1104879 15567851

[B90] Wick R. R. Judd L. M. Holt K. E. (2019). Performance of neural network basecalling tools for Oxford Nanopore sequencing. *Genome Biol.* 20:129. 10.1186/s13059-019-1727-y 31234903 PMC6591954

[B91] Wicker T. Sabot F. Hua-Van A. Bennetzen J. L. Capy P. Chalhoub B. (2007). A unified classification system for eukaryotic transposable elements. *Nat. Rev. Genet.* 8 973–982. 10.1038/nrg2165 17984973

[B92] Wu G. Fang Y.-Z. Yang S. Lupton J. R. Turner N. D. (2004). Glutathione metabolism and its implications for health. *J. Nut.* 134 489–492. 10.1093/jn/134.3.489 14988435

[B93] Xie C. Mao X. Huang J. Ding Y. Wu J. Dong S. (2011). KOBAS 2.0: a web server for annotation and identification of enriched pathways and diseases. *Nucleic Acids Res.* 39(Suppl. 2) W316–W322. 10.1093/nar/gkr483 21715386 PMC3125809

[B94] Xu Z. Wang H. (2007). LTR_FINDER: an efficient tool for the prediction of full-length LTR retrotransposons. *Nucleic Acids Res.* 35 W265–W268. 10.1093/nar/gkm286 17485477 PMC1933203

[B95] Zhang S. Skerker J. M. Rutter C. D. Maurer M. J. Arkin A. P. Rao C. V. (2016). Engineering *Rhodosporidium toruloides* for increased lipid production. *Biotechnol. Bioeng.* 113 1056–1066. 10.1002/bit.25864 26479039

