# Supplementary material for: Genome-Wide Transcriptional Changes of Rhodosporidium kratochvilovae at Low Temperature
Source: Front Microbiol. 2021 Sep 16;12:727105. doi: 10.3389/fmicb.2021.727105 (PMC8481953; doi:10.3389/fmicb.2021.727105)
Supplement: Supplementary file 1 [file Data_Sheet_1.docx]

# Additional materials

Fig.S1 Result of principal component analysis (PCA) of all the sample


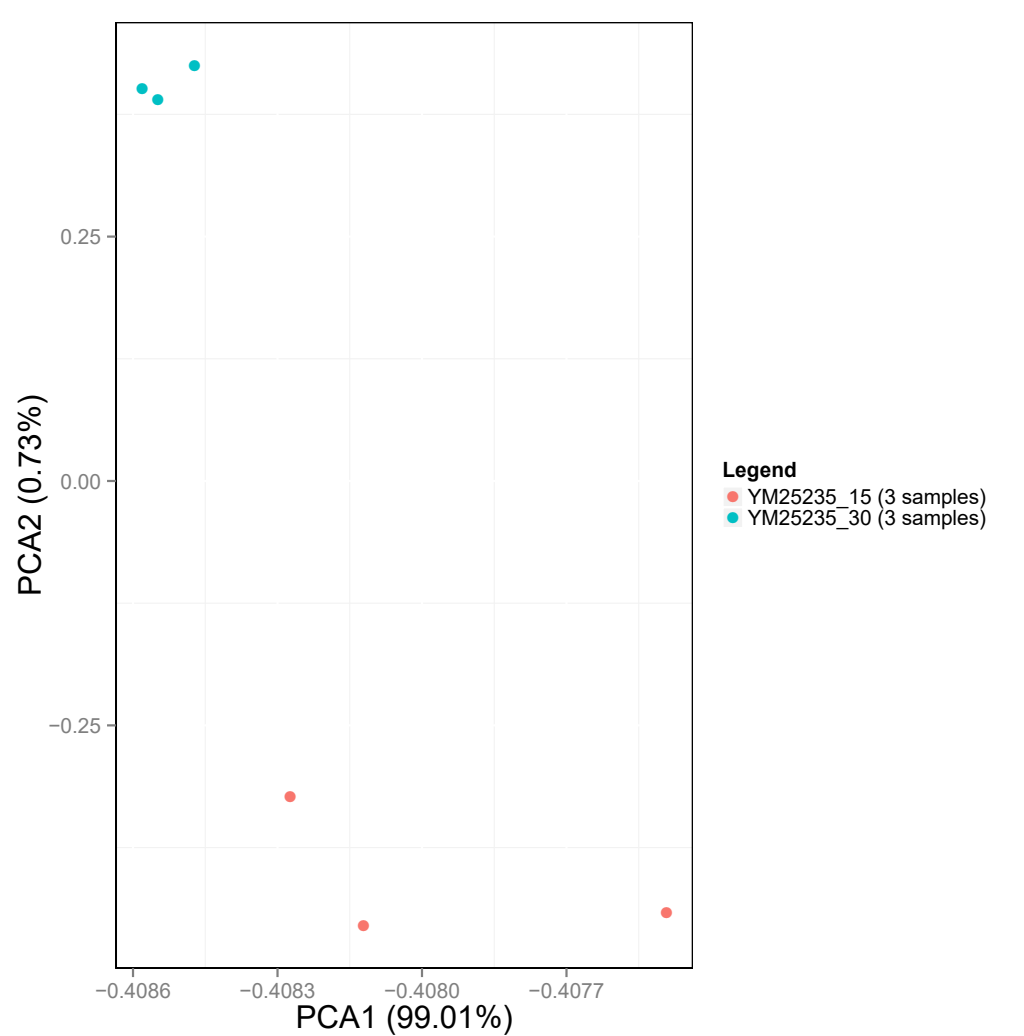


Fig.S2 Upregulated and downregulated DEGs in enriched pathways


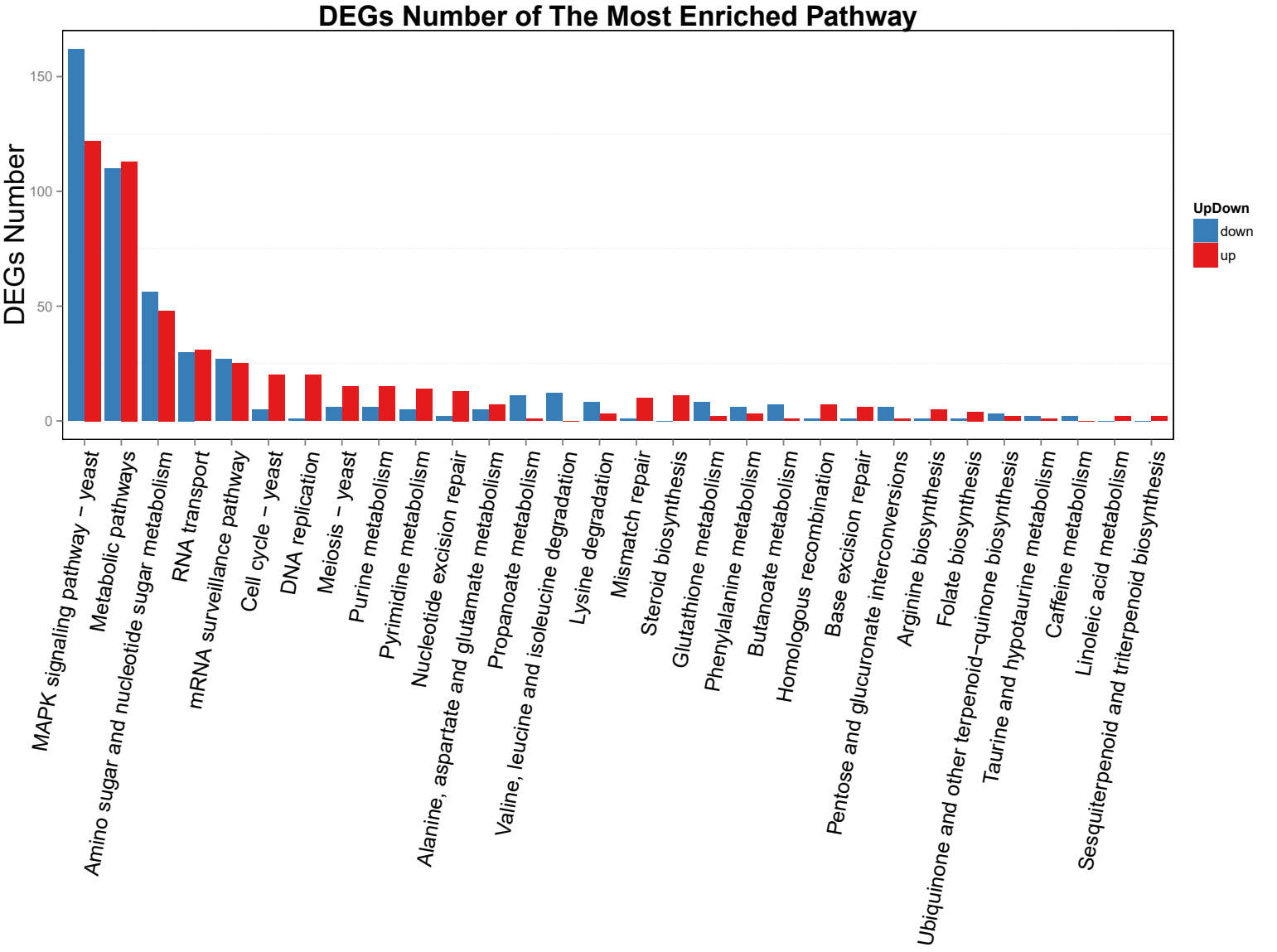


Table S1 Specific primers used in the qPCR analysis.

| Gene ID | Gene symbol | Sequence (5'-3') F | Sequence (5'-3') R |
| --- | --- | --- | --- |
| EVM0001337 | *AAD* | AAGGAGGAGTCGTTCAAG | CTCGTTCTGGTAGTTGTTC |
| EVM0007530 | *LigA* | TACATCATTCCTCCTGCTAAT | GTATCCGTCGTTGCTGAC |
| EVM0004509 | *Adh* | GTCAAGGACTGGATCAACAA | TCAGGCGTCGTCTTGTAG |
| EVM0008180 | *ALS* | AAGCATACACGCACCTGA | AATGACGCTCTCGTTACTG |
| EVM0001603 | *GCH2* | CTCGGCGTAAAGAAGATC | TCTCATACCTGCGAACTA |
| EVM0002833 | *4CCL* | TCTCGCTTCAAGACGCTC | GCACCTTGTGGTCGTTCA |
| EVM0000198 | *rad17* | CTGCTTCAATTCGTCACC | CGCTTGTTGTAGAGGACTT |
| EVM0008132 | *SLP* | CCTGAATACGGTCTCTACT | AATGGTGACATCCTTGGA |
| EVM0002161 | *ICL* | GACGAGAACCAGATGAAGAG | GGCGAGGGTGATGAACTG |
| EVM0008084 | *ppg4* | AACAACTACTTTCAAGGG | CTTCATCATGCTCTTCCA |
| EVM0001915 | *hisG* | AACAACTACTTTCAAGGG | CTTCATCATGCTCTTCCA |
| EVM0001854 | *GNAT* | CTCAAGGACACCTTCAAC | AAGAAGTAGGCAGAGAAC |
| EVM0003022 | *MFSmdf* | CTACAAGAGCGTGAGCAT | AGAGATAGCAGTCGATAATGT |
| EVM0006464 | *FCY* | ATCTGGATGACGATGAAC | AATGATGAGGAACGAGTC |
| EVM0008125 | *RTK* | ACCAACGAACAGCAGATG | AAGAAGACCATGACAGCAA |

Table S2 The number of genes annotated with each database.

| Anno database | Annotated number | 100<=length<300 | length>= 300 |
| --- | --- | --- | --- |
| GO Annotation | 2288 | 566 | 1675 |
| KEGG Annotation | 3143 | 790 | 2294 |
| KOG Annotation | 4602 | 1027 | 3522 |
| Pfam Annotation | 5796 | 1372 | 4349 |
| SwissProt Annotation | 4963 | 1088 | 3809 |
| TrEMBL Annotation | 7862 | 1989 | 5746 |
| Nr Annotation | 7859 | 1988 | 5744 |
| All Annotated | 7907 | 2003 | 5777 |

Table S3 The pathways enriched by DEGs between low temperature and control

| Number | ID | Pathway | DEG number | Q value |
| --- | --- | --- | --- | --- |
| 1 | ko03030 | DNA replication | 21 | 8.39E-07 |
| 2 | ko00100 | Steroid biosynthesis | 11 | 3.10E-05 |
| 3 | ko00640 | Propanoate metabolism | 12 | 7.87E-03 |
| 4 | ko03430 | Mismatch repair | 11 | 1.21E-02 |
| 5 | ko03420 | Nucleotide excision repair | 15 | 2.11E-02 |
| 6 | ko00520 | Amino sugar and nucleotide sugar metabolism | 104 | 2.11E-02 |
| 7 | ko01100 | Metabolic pathways | 223 | 3.04E-02 |
| 8 | ko00650 | Butanoate metabolism | 8 | 3.04E-02 |
| 9 | ko04011 | MAPK signaling pathway-yeast | 284 | 3.04E-02 |
| 10 | ko04113 | Meiosis-yeast | 21 | 3.04E-02 |
| 11 | ko00130 | Ubiquinone and other terpenoid-quinone biosynthesis | 5 | 4.88E-02 |
| 12 | ko00250 | Alanine, aspartate and glutamate metabolism | 12 | 4.88E-02 |
| 13 | ko04111 | Cell cycle-yeast | 25 | 4.88E-02 |
| 14 | ko00040 | Pentose and glucuronate interconversions | 7 | 6.03E-02 |
| 15 | ko03440 | Homologous recombination | 8 | 1.25E-01 |
| 16 | ko00360 | Phenylalanine metabolism | 9 | 1.25E-01 |
| 17 | ko00909 | Sesquiterpenoid and triterpenoid biosynthesis | 2 | 1.25E-01 |
| 18 | ko00232 | Caffeine metabolism | 2 | 1.25E-01 |
| 19 | ko00480 | Glutathione metabolism | 10 | 1.25E-01 |
| 20 | ko00240 | Pyrimidine metabolism | 19 | 1.25E-01 |
| 21 | ko00280 | Valine, leucine and isoleucine degradation | 12 | 1.44E-01 |
| 22 | ko00220 | Arginine biosynthesis | 6 | 1.49E-01 |
| 23 | ko03410 | Base excision repair | 7 | 2.56E-01 |
| 24 | ko00591 | Linoleic acid metabolism | 2 | 2.61E-01 |
| 25 | ko00230 | Purine metabolism | 21 | 2.61E-01 |
| 26 | ko03015 | mRNA surveillance pathway | 52 | 3.91E-01 |
| 27 | ko00790 | Folate biosynthesis | 5 | 4.66E-01 |
| 28 | ko00430 | Taurine and hypotaurine metabolism | 3 | 5.30E-01 |
| 29 | ko00310 | Lysine degradation | 11 | 5.76E-01 |
| 30 | ko03013 | RNA transport | 61 | 6.23E-01 |
| 31 | ko00513 | Various types of N-glycan biosynthesis | 6 | 6.23E-01 |
| 32 | ko00910 | Nitrogen metabolism | 4 | 6.90E-01 |
| 33 | ko00906 | Carotenoid biosynthesis | 1 | 8.58E-01 |
| 34 | ko00254 | Aflatoxin biosynthesis | 1 | 8.58E-01 |
| 35 | ko01130 | Biosynthesis of antibiotics | 39 | 8.58E-01 |
| 36 | ko01040 | Biosynthesis of unsaturated fatty acids | 4 | 8.58E-01 |
| 37 | ko00760 | Nicotinate and nicotinamide metabolism | 4 | 8.58E-01 |
| 38 | ko00561 | Glycerolipid metabolism | 6 | 8.58E-01 |
| 39 | ko00400 | Phenylalanine, tyrosine and tryptophan biosynthesis | 5 | 8.58E-01 |
| 40 | ko00410 | beta-Alanine metabolism | 6 | 8.66E-01 |
| 41 | ko00592 | alpha-Linolenic acid metabolism | 2 | 8.66E-01 |
| 42 | ko00740 | Riboflavin metabolism | 3 | 8.66E-01 |
| 43 | ko00670 | One carbon pool by folate | 3 | 8.66E-01 |
| 44 | ko00072 | Synthesis and degradation of ketone bodies | 1 | 9.04E-01 |
| 45 | ko00660 | C5-Branched dibasic acid metabolism | 1 | 9.04E-01 |
| 46 | ko00062 | Fatty acid elongation | 2 | 9.04E-01 |
| 47 | ko00750 | Vitamin B6 metabolism | 2 | 9.04E-01 |
| 48 | ko00730 | Thiamine metabolism | 3 | 9.43E-01 |
| 49 | ko00350 | Tyrosine metabolism | 5 | 9.43E-01 |
| 50 | ko00780 | Biotin metabolism | 2 | 9.43E-01 |
| 51 | ko00071 | Fatty acid degradation | 6 | 9.43E-01 |
| 52 | ko00564 | Glycerophospholipid metabolism | 7 | 9.43E-01 |
| 53 | ko04979 | Cholesterol metabolism | 1 | 9.43E-01 |
| 54 | ko00261 | Monobactam biosynthesis | 1 | 9.43E-01 |
| 55 | ko00053 | Ascorbate and aldarate metabolism | 2 | 9.64E-01 |
| 56 | ko03450 | Non-homologous end-joining | 2 | 9.64E-01 |
| 57 | ko04139 | Mitophagy-yeast | 9 | 9.87E-01 |
| 58 | ko01110 | Biosynthesis of secondary metabolites | 48 | 9.97E-01 |
| 59 | ko00290 | Valine, leucine and isoleucine biosynthesis | 2 | 9.97E-01 |
| 60 | ko01212 | Fatty acid metabolism | 5 | 9.97E-01 |
| 61 | ko01210 | 2-Oxocarboxylic acid metabolism | 5 | 1.00E+00 |
| 62 | ko04213 | Longevity regulating pathway-multiple species | 5 | 1.00E+00 |
| 63 | ko00620 | Pyruvate metabolism | 6 | 1.00E+00 |
| 64 | ko00562 | Inositol phosphate metabolism | 4 | 1.00E+00 |
| 65 | ko00051 | Fructose and mannose metabolism | 4 | 1.00E+00 |
| 66 | ko00510 | N-Glycan biosynthesis | 5 | 1.00E+00 |
| 67 | ko00380 | Tryptophan metabolism | 5 | 1.00E+00 |
| 68 | ko00300 | Lysine biosynthesis | 2 | 1.00E+00 |
| 69 | ko00330 | Arginine and proline metabolism | 4 | 1.00E+00 |
| 70 | ko04070 | Phosphatidylinositol signaling system | 3 | 1.00E+00 |
| 71 | ko00450 | Selenocompound metabolism | 1 | 1.00E+00 |
| 72 | ko01230 | Biosynthesis of amino acids | 15 | 1.00E+00 |
| 73 | ko00340 | Histidine metabolism | 2 | 1.00E+00 |
| 74 | ko00920 | Sulfur metabolism | 2 | 1.00E+00 |
| 75 | ko04146 | Peroxisome | 8 | 1.00E+00 |
| 76 | ko00770 | Pantothenate and CoA biosynthesis | 2 | 1.00E+00 |
| 77 | ko04392 | Hippo signaling pathway-multiple species | 1 | 1.00E+00 |
| 78 | ko00565 | Ether lipid metabolism | 1 | 1.00E+00 |
| 79 | ko00500 | Starch and sucrose metabolism | 3 | 1.00E+00 |
| 80 | ko00630 | Glyoxylate and dicarboxylate metabolism | 3 | 1.00E+00 |
| 81 | ko03008 | Ribosome biogenesis in eukaryotes | 10 | 1.00E+00 |
| 82 | ko00061 | Fatty acid biosynthesis | 1 | 1.00E+00 |
| 83 | ko00052 | Galactose metabolism | 1 | 1.00E+00 |
| 84 | ko00010 | Glycolysis/Gluconeogenesis | 5 | 1.00E+00 |
| 85 | ko03022 | Basal transcription factors | 3 | 1.00E+00 |
| 86 | ko00900 | Terpenoid backbone biosynthesis | 1 | 1.00E+00 |
| 87 | ko00270 | Cysteine and methionine metabolism | 4 | 1.00E+00 |
| 88 | ko00260 | Glycine, serine and threonine metabolism | 4 | 1.00E+00 |
| 89 | ko03020 | RNA polymerase | 3 | 1.00E+00 |
| 90 | ko00860 | Porphyrin and chlorophyll metabolism | 1 | 1.00E+00 |
| 91 | ko00460 | Cyanoamino acid metabolism | 1 | 1.00E+00 |
| 92 | ko04144 | Endocytosis | 53 | 1.00E+00 |
| 93 | ko00600 | Sphingolipid metabolism | 1 | 1.00E+00 |
| 94 | ko00030 | Pentose phosphate pathway | 1 | 1.00E+00 |
| 95 | ko04130 | SNARE interactions in vesicular transport | 1 | 1.00E+00 |
| 96 | ko04145 | Phagosome | 3 | 1.00E+00 |
| 97 | ko00680 | Methane metabolism | 1 | 1.00E+00 |
| 98 | ko01200 | Carbon metabolism | 9 | 1.00E+00 |
| 99 | ko04120 | Ubiquitin mediated proteolysis | 4 | 1.00E+00 |
| 100 | ko03018 | RNA degradation | 4 | 1.00E+00 |
| 101 | ko00563 | Glycosylphosphatidylinositol (GPI)-anchor biosynthesis | 1 | 1.00E+00 |
| 102 | ko04141 | Protein processing in endoplasmic reticulum | 5 | 1.00E+00 |
| 103 | ko00970 | Aminoacyl-tRNA biosynthesis | 1 | 1.00E+00 |
| 104 | ko03040 | Spliceosome | 8 | 1.00E+00 |
| 105 | ko04138 | Autophagy-yeast | 2 | 1.00E+00 |
| 106 | ko03010 | Ribosome | 6 | 1.00E+00 |
| 107 | ko00190 | Oxidative phosphorylation | 1 | 1.00E+00 |
